# SOCIAL SUPPORT AND STRAIN AS PREDICTORS OF MULTIMORBIDITY FOLLOWING A MARITAL TRANSITION

**DOI:** 10.1093/geroni/igac059.2136

**Published:** 2022-12-20

**Authors:** Emily Denning, Jason Newsom, Anda Botoseneanu, Heather Allore, Corey Nagel, David Dorr, Ana Quiñones

**Affiliations:** Portland State University, Portland, Oregon, United States; Portland State University, Portland, Oregon, United States; University of Michigan Dearborn, Dearborn, Michigan, United States; Yale University, Guilfrod, Connecticut, United States; University of Arkansas for Medical Sciences, Little Rock, Arkansas, United States; Oregon Health & Science University, Portland, Oregon, United States; Oregon Health and Science University, Portland, Oregon, United States

## Abstract

Marital transitions (MTs; widowhood or divorce) are stressful events that impact the health of older adults. This study examined the impact of social support and social strain on multimorbidity trajectories using data from the Health and Retirement Study (HRS). Participants were 377 adults age 50+ with a single MT between years 2006 and 2016. We used piecewise growth curve modeling to investigate whether social support and strain from one’s spouse, children, family, or friends, measured prior to transition, predicted trajectories of chronic conditions (count of 8 conditions: hypertension, diabetes, cancer, lung disease, heart disease, stroke, arthritis, and cognitive impairment) following MT. Covariates included sex, age, education, race/ethnicity, and wealth. On average, chronic conditions were increasing before MT, B = .172, SE = .021, p < .001, and after MT, B = .211, SE = .031, p <.001. Participants had an average of 2.2 chronic conditions at MT. Spousal support prior to MT was associated with fewer chronic conditions at MT, B = -.863, SE = .427, p = .043, whereas support and social strain from friends were each associated with more chronic conditions at MT (support: B = .772, SE = .354, p = .025; strain: B = 1.288, SE = .387, p = .001). Support from children was positively associated with more chronic conditions following MT, B = .212, SE = .084, p = .011, which may reflect adult children providing support in response to parental health decline.

